# Differential Regulation of Caspase-1 Activation, Pyroptosis, and Autophagy via Ipaf and ASC in *Shigella*-Infected Macrophages

**DOI:** 10.1371/journal.ppat.0030111

**Published:** 2007-08-10

**Authors:** Toshihiko Suzuki, Luigi Franchi, Claudia Toma, Hiroshi Ashida, Michinaga Ogawa, Yuko Yoshikawa, Hitomi Mimuro, Naohiro Inohara, Chihiro Sasakawa, Gabriel Nuñez

**Affiliations:** 1 Division of Bacterial Pathogenesis, Graduate School of Medicine, University of the Ryukyus, Okinawa, Japan; 2 Department of Pathology and Comprehensive Cancer Center, University of Michigan Medical School, Ann Arbor, Michigan, United States of America; 3 Department of Microbiology and Immunology, International Research Center for Infectious Diseases, Institute of Medical Science, University of Tokyo, Tokyo, Japan; 4 Department of Infectious Disease Control, International Research Center for Infectious Diseases, Institute of Medical Science, University of Tokyo, Tokyo, Japan; 5 Core Research for Evolutional Science and Technology, Japan Science and Technology Agency, Kawaguchi, Japan; University of Washington, United States of America

## Abstract

*Shigella* infection, the cause of bacillary dysentery, induces caspase-1 activation and cell death in macrophages, but the precise mechanisms of this activation remain poorly understood. We demonstrate here that caspase-1 activation and IL-1β processing induced by *Shigella* are mediated through Ipaf, a cytosolic pattern-recognition receptor of the nucleotide-binding oligomerization domain (NOD)-like receptor (NLR) family, and the adaptor protein apoptosis-associated speck-like protein containing a C-terminal caspase recruitment domain (ASC). We also show that Ipaf was critical for pyroptosis, a specialized form of caspase-1-dependent cell death induced in macrophages by bacterial infection, whereas ASC was dispensable. Unlike that observed in *Salmonella* and *Legionella,* caspase-1 activation induced by *Shigella* infection was independent of flagellin. Notably, infection of macrophages with *Shigella* induced autophagy, which was dramatically increased by the absence of caspase-1 or Ipaf, but not ASC. Autophagy induced by *Shigella* required an intact bacterial type III secretion system but not VirG protein, a bacterial factor required for autophagy in epithelial-infected cells. Treatment of macrophages with 3-methyladenine, an inhibitor of autophagy, enhanced pyroptosis induced by *Shigella* infection, suggesting that autophagy protects infected macrophages from pyroptosis. Thus, Ipaf plays a critical role in caspase-1 activation induced by *Shigella* independently of flagellin. Furthermore, the absence of Ipaf or caspase-1, but not ASC, regulates pyroptosis and the induction of autophagy in *Shigella*-infected macrophages, providing a novel function for NLR proteins in bacterial–host interactions.

## Author Summary

*Shigella* are bacterial pathogens that are the cause of bacillary dysentery known as shigellosis. A crucial aspect of the propensity of *Shigella* to cause diseases lies in its ability to invade the cytoplasm of epithelial cells as well as macrophages. The bacterial invasion of macrophages induces pyroptosis, the proinflammatory cell death associated with caspase-1 activation. Activated caspase-1 then cleaves and activates prointerleukin (proIL)-1β and proIL-18, which are proinflammatory cytokines involved in host inflammatory responses. However, the precise mechanisms of caspase-1 activation induced by *Shigella* infection remain poorly understood. Ipaf, a cytosolic pattern-recognition receptor of the nucleotide-binding oligomerization domain (NOD)-like receptor (NLR) family, is a crucial host factor that activates caspase-1 through the sensing of flagellin produced by some bacteria, such as *Salmonella* or *Legionella*. We discovered that Ipaf and the adaptor protein ASC are required for caspase-1 activation induced by non-flagellated *Shigella* infection. Thus, Ipaf and ASC mediate caspase-1 activation by sensing an unknown bacterial factor, but not flagellin. Autophagy, a cellular system for eliminating intracellular pathogens, was dramatically enhanced in *Shigella*-infected macrophages by the absence of caspase-1 or Ipaf, but not ASC. The inhibition of autophagy promoted *Shigella*-induced cell death, suggesting that autophagy protects infected macrophages from pyroptosis. This study provides evidence that in *Shigella*-infected macrophages, autophagy is inhibited by Ipaf and caspase-1, but positively regulated by ASC, providing a novel function for NLR proteins in bacterial–host interactions.

## Introduction

An effective immune response against microbial pathogens relies on the ability of the host to sense the presence of the infectious agent as well as the ability to destroy the invading pathogen. The presence of infection is detected through pathogen recognition molecules that sense unique microbial components called pathogen-associated molecular patterns (PAMPs) [1,2]. The recognition of bacterial PAMPs is mediated by several host molecules, including Toll-like receptors (TLRs) that are present on the cell surface and endosomal compartments, as well as nucleotide-binding oligomerization domain (NOD)-like receptors (NLRs) that sense the presence of PAMPs in the cytosol [1,2]. The NLR protein family contains more than 20 members, including Nod1, Nod2, cryopyrin (also called as Nalp3), Nalp1, and Ipaf. NLR proteins contain C-terminal leucine-rich repeats that are linked to microbial recognition, a centrally located NOD domain that mediates oligomerization, and an N-terminal effector domain that includes caspase-activation and recruitment domain or pyrin domain [3–5]. Cryopyrin and Ipaf have been implicated in caspase-1 activation and interleukin (IL)-1β processing induced by TLR agonists, gout-associated uric acid crystals, and specific bacterial infection [6–9]. Ipaf has been shown to mediate caspase-1 activation, IL-1β processing, and caspase-1-dependent cytotoxicity induced by intracellular *Salmonella* or *Legionella* [10–14]. Caspase-1 activation and IL-1β processing induced through Nalp3 or Ipaf also required the adaptor protein ASC (apoptosis-associated speck-like protein containing a C-terminal caspase recruitment domain), which is thought to be important for the formation of the inflammasome, a multiprotein complex that mediates caspase-1 activation [6,10,15]. NLR proteins such as cryopyrin and Ipaf play a crucial role in processing mature IL-1β (also IL-18), which are important inflammatory cytokines in host defense against infection and pathogenesis of inflammatory disorders [16–18].

*Shigella* are highly adapted human pathogens that cause bacillary dysentery (also referred to as shigellosis). The prominent pathogenic feature of *Shigella* is their ability to invade a variety of host cells, including epithelial cells, macrophages, and dendritic cells, which leads to severe inflammatory responses in intestinal tissue. Internalized *Shigella* multiply in the cytoplasm of epithelial cells and induce actin polymerization at one pole of the bacterium, allowing intracellular bacteria to move within the cytoplasm and to spread into adjacent epithelial cells [19,20]. *Shigella* also invade resident macrophages in the intestinal tissue, and the bacteria escape from the phagosome into the cytosol. Infected macrophages undergo caspase-1-mediated cell death, termed pyroptosis, which is a newly identified pathway of programmed cell death associated with an inflammatory response that is accompanied by plasma membrane permeability and nuclear condensation [21–23]. In the macrophage cytosol, *Shigella* induce pyroptosis through activation of caspase-1 that is dependent on IpaB, a protein secreted via the type III secretion system (TTSS) or by lipopolysaccharide (LPS) moiety released from the bacteria, leading to the processing and secretion of IL-1β [23,24]. As a result, pro-inflammatory chemokines and cytokines produced by the macrophages and epithelial cells infected with *Shigella* elicit strong inflammation in the intestinal tissue.

Flagellin, a major subunit of the flagellum, is a prerequisite for pyroptosis and caspase-1 activation of infected macrophages with *Salmonella* and *Legionella* [11,12,14,25,26]. In the case of *Salmonella,* Ipaf has been shown to be essential for caspase-1 activation and pyroptosis by sensing flagellin in the cytosol [11,12]. In this study, we examined the mechanisms that regulate caspase-1 activation or cell death induced by *Shigella* in macrophages. Surprisingly, we find that Ipaf mediates caspase-1 activation and cell death independently of flagellin in *Shigella*-infected macrophages. In addition, autophagy was induced by *Shigella* infection, and this process was negatively regulated by Ipaf and caspase-1, but not ASC.

## Results

### *Shigella* Infection Induces Caspase-1 Activation and Pyroptosis in Macrophages Independently of Flagellin

**Figure 1:**
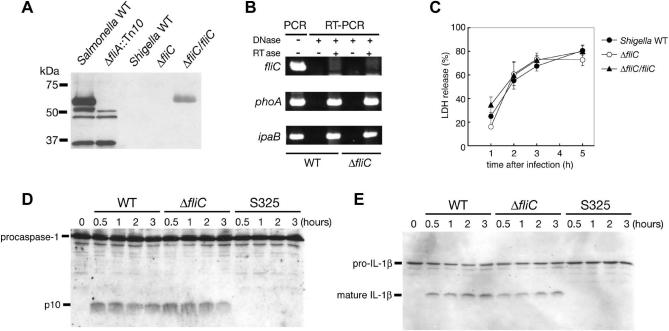
Flagellin-Independent Caspase-1 Activation and Pyroptosis in *Shigella*-Infected Macrophages (A) Immunoblot for FliC expression. Bacterial whole cell lysates were loaded from S. typhimurium strains: wild-type (WT), flagellar mutant (*fliA*::Tn*10*); or S. flexneri strains: WT, flagellin mutant (*ΔfliC*), flagellin overexpressor (*ΔfliC*/*fliC*). (B) RT-PCR for *fliC* mRNA expression in *Shigella*. As the controls, *phoA* or *ipaB* expression was examined. (C) LDH release from BMMs infected with *Shigella* WT, *ΔfliC,* or *ΔfliC*/*fliC*. Error bars represent mean ± SD. (D) Activation of caspase-1, assessed by immunoblot for the processed p10 fragment after infection with *Shigella* WT, Δ*fliC,* or TTSS mutant (S325). (E) Processing of IL-1β, assessed by immunoblot. Results are representative of three experiments.

Flagellin is required for pyroptosis and caspase-1 activation of macrophages infected with *Salmonella* and *Legionella* [11,12,14,25,26]. On the other hand, Shigella flexneri strains are not motile and are non-flagellated bacteria. To examine whether S. flexneri express flagellin, genome sequence information from the strains 2457T [27] and 301 [28] was compared in silico with that of S. enterica serovar typhimurium LT2 [29]. Forty-two genes are associated with flagella formation in *Salmonella,* including *fliC* and *fljB,* two genes that encode flagellin proteins [30]. Several genes that are known to be essential for flagellar assembly in *Salmonella* were absent in *Shigella*. Eight genes (*flgC, F, K, L, flhD, fliF, J,* and *P*) were absent in the 2457T strain, and seven genes (*flgC, D, F, K, L, flhD,* and *fliF*) were deleted in the 301 *Shigella* strain. In particular, we found that *flhD,* the master regulatory gene that controls the transcription of flagellar genes [31], was not present in S. flexneri. These results suggest that both flagellar assembly and flagellin expression is deficient in *Shigella*. Consistent with this notion, *Salmonella,* but not *Shigella,* expressed FliC by western blot analysis with anti-FliC antibody (Figure 1A). In addition, the expression of the *Shigella fliC* gene was not detected by RT-PCR analysis (Figure 1B). However, the open reading frame of the *Shigella* flagellin gene (*fliC*) was intact in that expression of flagellin was induced under the inducible promoter after *fliC* plasmid complementation (Figure 1A).

We next examined the involvement of FliC in *Shigella*-induced pyroptosis. Mouse bone marrow–derived macrophages (BMMs) were infected with wild-type *Shigella,* Δ*fliC* mutant, or Δ*fliC* mutant strain complemented with *fliC* (Δ*fliC*/*fliC*), and cell death was examined by lactose dehydrogenase (LDH) release in infected cells. All the strains examined had similar kinetics of LDH release (Figure 1C). Thus, FliC expression is not essential for pyroptosis induction in *Shigella*-infected BMMs. Moreover, infection of BMMs with wild-type *Shigella* and Δ*fliC* mutant, but not TTSS-deficient mutant (S325, *mxiA*::Tn*5*), induced caspase-1 activation and caspase-1-mediated IL-1β processing (Figure 1D and 1E). These results indicate that an intact TTSS, but not flagellin, is required for caspase-1 activation and IL-1β processing in *Shigella*-infected BMMs.

### Critical Role of the Ipaf-ASC Inflammasome for *Shigella*-Induced Caspase-1 Activation

**Figure 2:**
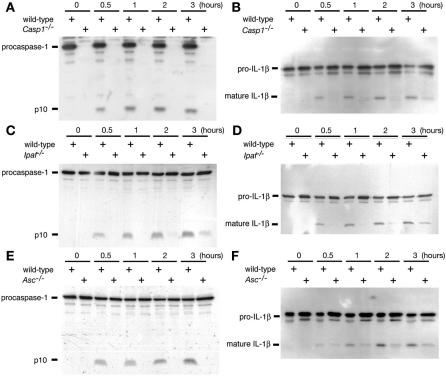
Ipaf and ASC Are Required for *Shigella*-Induced Caspase-1 and IL-1β Processing Wild-type, caspase-1-deficient, Ipaf-deficient, or ASC-deficient BMMs were infected with *Shigella* WT. Immunoblot for caspase-1 p10 (A, C, and E) and for IL-1β (B, D, and F). Blots are representative of three experiments.

Ipaf and ASC are required for caspase-1 activation in *Salmonella*-infected macrophages [10–12]. To gain insight into the molecular mechanism responsible for caspase-1 activation induced by *Shigella* infection, we analyzed caspase-1 activation in infected BMMs isolated from wild-type, caspase-1-deficient, Ipaf-deficient, or ASC-deficient mice (Figure 2). After infection, the processed p10 fragment from procaspase-1 was detected in wild-type BMMs, but this proteolytic cleavage was impaired in Ipaf-deficient and ASC-deficient BMMs (Figure 2C and 2E). Similarly, IL-1β processing was impaired in Ipaf-deficient and ASC-deficient BMMs, but not in wild-type BMMs (Figure 2D and 2F). At 2 or 3 h post infection, low levels of mature IL-1β were detected after infection of Ipaf-deficient BMMs, and at an earlier time in ASC-deficient cells, suggesting that in addition to caspase-1, other proteases can contribute to proIL-1β cleavage. This is consistent with detection of low levels of mature IL-1β in caspase-1-deficient BMMs at later time points (Figure 2B). The presence of residual proIL-1β cleavage at earlier time points in ASC-deficient macrophages may reflect increased cell death in response to *Shigella* when compared to Ipaf- and caspase-1-deficient macrophages (see below). It is also possible that other NLRs may contribute to caspase-1 activation in Ipaf- or ASC-deficient BMMs during *Shigella* infection. These events are reminiscent of the *Salmonella* system in which at high multiplicity of infection (MOI), there is residual IL-1β secretion that is induced independently of cytosolic flagellin and, presumably, of Ipaf [12]. The uptake of bacteria was similar in wild-type, caspase-1-deficient, Ipaf-deficient, and ASC-deficient BMMs (unpublished data), suggesting that deficient internalization of *Shigella* was not responsible for the phenotype. These results indicate that Ipaf and ASC are important for *Shigella*-inducing caspase-1 activation and subsequent IL-1β processing. Furthermore, unlike in *Salmonella,* the activation of the Ipaf-ASC inflammasome is independent of flagellin.

### Ipaf, but Not ASC, Is Involved in Induction of Pyroptosis in *Shigella*-Infected Macrophages

**Figure 3:**
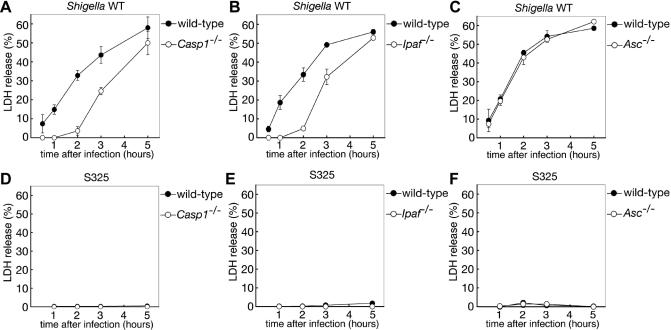
The Early Phase of Pyroptosis Induced by *Shigella* Is Dependent on Ipaf but Not ASC Caspase-1-deficient (A and D), Ipaf-deficient (B and E), or ASC-deficient BMMs (C and F) were infected with *Shigella* WT (A–C) or TTSS mutant S325 (D–F). LDH released from infected BMMs were quantified at the indicated time. Error bars represent mean ± SD.

BMMs derived from Ipaf-deficient mice, but not ASC-deficient mice, are resistant to caspase-1-dependent *Salmonella*-induced pyroptosis [10–12]. To investigate the role of Ipaf and ASC in *Shigella*-inducing pyroptosis, we analyzed LDH release from infected wild-type, Ipaf-deficient, and ASC-deficient BMMs. We found that LDH release, a marker of pyroptosis, was abrogated in Ipaf-deficient BMMs within 2 h of *Shigella* infection when compared to wild-type BMMs, but the release was induced after 3 h of infection and by 5 h was comparable to that of wild-type BMMs (Figure 3B). The kinetics of LDH release induced by *Shigella* in Ipaf-deficient BMMs was similar to that in caspase-1-deficient BMMs (Figure 3A) [23]. However, the kinetics of LDH release induced by *Shigella* in ASC-deficient BMMs was indistinguishable from that observed in wild-type BMMs (Figure 3C), suggesting that ASC is not important in cell death induced by *Shigella,* despite ASC being required for caspase-1 activation (Figure 2E). Notably, Ipaf- and ASC-deficient BMMs did not undergo rapid LDH release when infected with the *Shigella* TTSS mutant (Figure 3E and 3F), indicating that an intact TTSS is required for Ipaf-dependent induction of pyroptosis. Thus, Ipaf, but not ASC, is required for the rapid pyroptotic response induced by *Shigella* infection in BMMs. Furthermore, caspase-1 activation may be dispensable for pyroptotic cell death induced by *Shigella* infection in the absence of ASC.

### The Absence of Caspase-1 Promotes Autophagosome Maturation in *Shigella-*Infected BMMs

**Figure 4:**
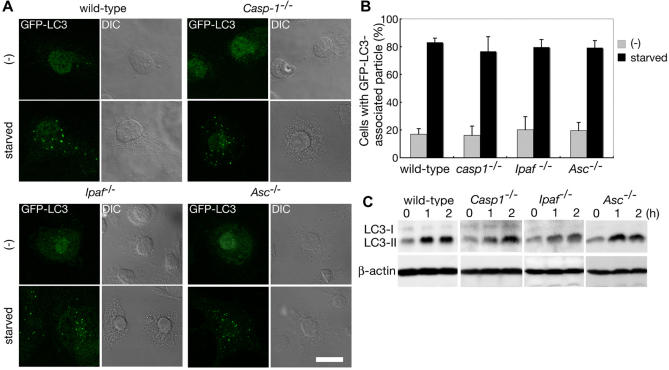
Autophagy Induced by Amino Acid Starvation or Rapamycin Treatment Is Independent of Caspase-1, Ipaf, and ASC (A) GFP-LC3-expressing wild-type BMMs and caspase-1-deficient, Ipaf-deficient, or ASC-deficient BMMs were incubated in amino acid–starved conditions for 2 h. GFP fluorescence and differential interference contrast (DIC) were shown separately. Scale bar = 10 μm. (B) BMMs with GFP-LC3-associated autophagosomal particles were quantified. Error bars represent mean ± SD. (C) Endogenous LC3-I to LC3-II conversion upon rapamycin treatment was analyzed by western blotting using anti-LC3 antibody. The immunoblot for β-actin was indicated as an internal control.

**Figure 5:**
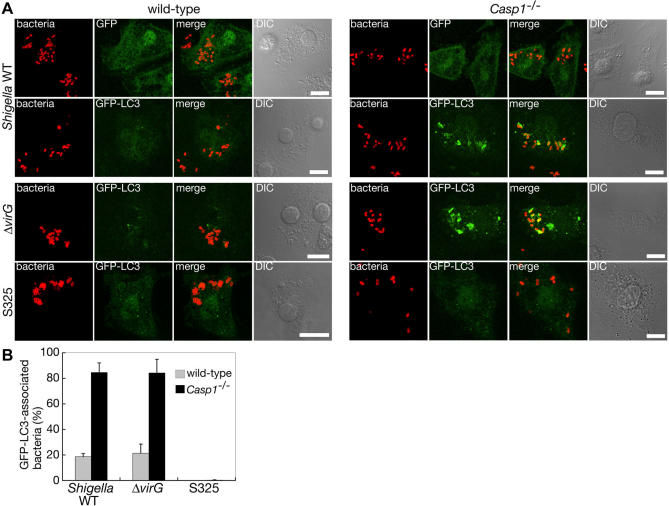
Caspase-1 Deficiency Promotes Autophagosome Maturation in Macrophages Infected with *Shigella* Independently of VirG Protein GFP-LC3-expressing wild-type or caspase-1-deficient BMMs were infected with *Shigella* WT, Δ*virG,* or TTSS mutant S325. As a control, GFP alone expressing wild-type or caspase-1-deficient BMMs were infected with *Shigella* WT. (A) At 30 min after infection, the infected cells were immunostainted with Cy5-labeled anti- *Shigella* LPS antibody (colored red) and examined using a confocal microscope. The merged image with Cy5 bacteria and GFP fluorescence, and differential interference contrast (DIC) were also shown. Scale bars = 10 μm. (B) GFP-LC3-associated intracellular bacteria were quantified. Error bars represent mean ± SD.

**Figure 6:**
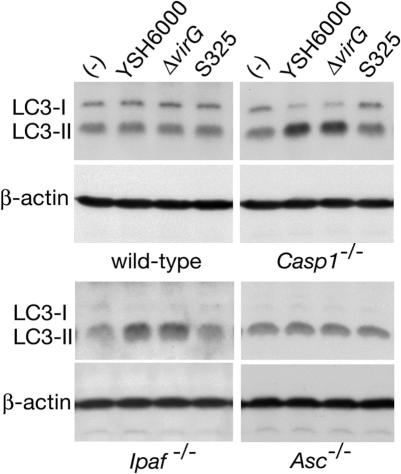
Endogenous LC3-I to LC3-II Conversion in BMMs Infected by *Shigella* Wild-type, caspase-1-deficient, Ipaf-deficient, or ASC-deficient BMMs were infected with *Shigella* WT, Δ*virG,* or TTSS mutant S325. At 30 min after infection, the total lysates of BMMs were prepared and analyzed by western blotting using anti-LC3 antibody. The immunoblot for β-actin was indicated as an internal control.

Autophagy is induced by diverse death stimuli, including that associated with caspase-independent death, but the regulation of autophagy triggered by bacterial infection is poorly understood [32]. In pathogen-infected cells, autophagy appears to function as a host defense mechanism that can be subverted by certain virulent bacteria to enhance their intracellular replication [33–37]. To study the role of Ipaf and ASC in autophagy, we first examined whether autophagy is induced in *Shigella*-infected BMMs. To assess autophagy, wild-type and caspase-1-deficient BMMs were transfected with GFP-LC3 (Atg8, a marker protein of autophagy) using a retroviral vector, and the GFP-LC3 labeling pattern was visualized by fluorescence microscopy. Autophagy induced by amino acid starvation was not affected by caspase-1, because a similar number of GFP-LC3 aggregates that are typically associated with the formation of autophagosomal vesicles were observed in wild-type and caspase-1-, Ipaf-, and ASC-deficient BMMs (Figure 4A and 4B). As another approach, endogenous LC3-I to LC3-II conversion, which is an indicator of autophagosome maturation, was examined by western blotting after rapamycin treatment to induce autophagy [38–40]. Although LC3-II was more abundant than LC3-I in steady state in all BMMs, LC3 conversions (an increase of the amounts of LC3-II) were actually observed in wild-type and caspase-1-, Ipaf-, and ASC-deficient BMMs (Figure 4C), suggesting that rapamycin-induced autophagy is not affected by these genetic deficiencies. The cellular localization of GFP-LC3 was examined 30 min after infection with *Shigella,* since at this early time the membrane integrity of the majority of wild-type BMMs was retained (unpublished data). As shown in Figure 5A and 5B, nearly 20% of intracellular *Shigella* was associated with accumulated GFP-LC3 in wild-type BMMs, whereas the percentage increased to about 90% in caspase-1-deficient BMMs. No accumulation of GFP alone was observed in infected cells. These results indicate that the absence of caspase-1 promotes autophagosome maturation induced by *Shigella* infection. Interestingly, a large number of GFP-LC3-containing vesicles, which were not associated with bacteria, were observed in caspase-1-deficient BMMs infected with *Shigella* (Figure 5A), suggesting that endogenous autophagy was also activated during infection. The endogenous LC-I to LC3-II conversion was also detected by *Shigella* infection in caspase-1-deficient BMMs (Figure 6). In epithelial cells, internalized *Shigella* can escape from autophagy by secreting the IcsB effector, which interferes with the interaction of host Atg5 with bacterial surface protein VirG [41]. VirG is not only a bacterial factor essential for actin polymerization, but also a molecular target of host autophagy. Indeed, Δ*virG,* a *Shigella* mutant lacking VirG, did not induce GFP-LC3 accumulation in epithelial cells [41]. Notably, we found that unlike that observed in epithelial cells, Δ*virG* induced a similar level of GFP-LC3 aggregates as wild-type *Shigella* in BMMs (Figures 5A, 5B, and 6). These results indicate that factors other than VirG are involved in autophagy formation in caspase-1-deficient BMMs. In both wild-type and caspase-1-deficient BMMs, GFP-LC3 accumulation around the phagocytosed *Shigella* TTSS mutant (S325) and endogenous LC3 conversion were not induced (Figures 5A, 5B, and 6), suggesting that bacterial escape from the phagosome is required for autophagosome maturation in *Shigella*-infected BMMs.

### Ipaf, but Not ASC, Is Involved in Autophagy Induced by *Shigella* Infection

**Figure 7:**
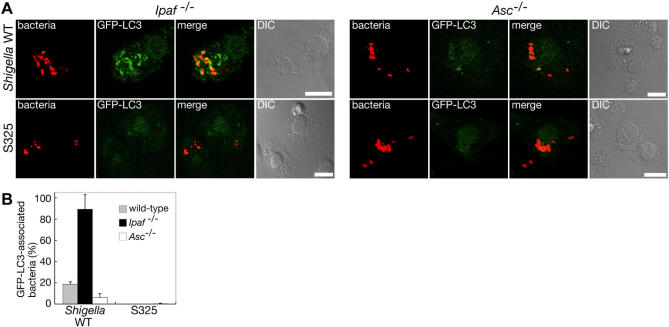
Differential Regulation of *Shigella*-Induced Autophagy by Ipaf and ASC GFP-LC3-expressing Ipaf-deficient or ASC-deficient BMMs were infected with *Shigella* WT or TTSS mutant. (A) At 30 min after infection, the infected cells were immunostainted with Cy5-labeled anti-*Shigella* LPS antibody (colored red) and examined using a confocal microscope. The merged image with Cy5 bacteria and GFP fluorescence, and differential interference contrast (DIC) were also shown. Scale bars = 10 μm. (B) GFP-LC3-associated intracellular bacteria were quantified. Error bars represent mean ± SD.

We next examined the role of Ipaf and ASC in autophagosome maturation in *Shigella*-infected BMMs. Similar to that observed in caspase-1-deficient BMMs, GFP-LC3 accumulation and endogenous LC3-I to LC3-II conversion were enhanced after *Shigella* infection in Ipaf-deficient BMMs when compared to wild-type cells (Figures 6, 7A, and 7B). Because caspase-1 activation induced by *Shigella* is deficient in caspase-1- and Ipaf-deficient BMMs, these results suggested that caspase-1 activation inhibits the induction of *Shigella*-induced autophagy. However, when ASC-deficient BMMs were infected with *Shigella,* the levels of autophagy associated with intracellular bacteria were similar to those observed in wild-type BMMs (Figures 6, 7A, and 6B), indicating that autophagosome maturation is not enhanced by ASC deficiency, even though caspase-1 activation is abrogated upon *Shigella* infection (Figure 2E). GFP-LC3-associated autophagic vesicles triggered by amino acid starvation and endogenous LC3-I to LC3-II conversion by rapamycin treatment were still induced in ASC-deficient BMMs, suggesting that the autophagic machinery is intact in the absence of ASC. Together with the results presented in Figure 3, these results indicate that Ipaf and ASC differentially regulate the induction of autophagy and suggest that autophagy in caspase-1- and Ipaf-deficient BMMs is associated with resistance to pyroptotic cell death.

### Inhibition of Autophagy with 3-Methyladenine Promotes Cell Death in *Shigella*-Infected Macrophages

**Figure 8:**
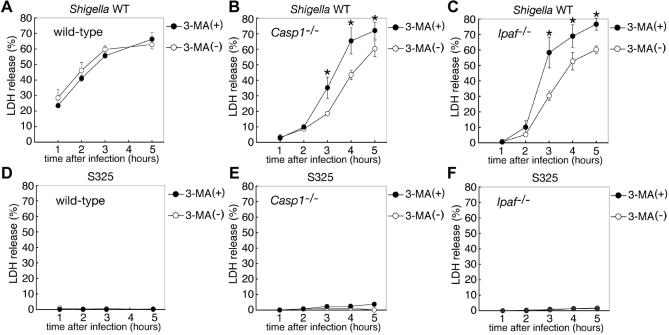
Autophagy Inhibitor 3-MA Enhances Caspase-1-Independent or Ipaf-Independent Cell Death of Macrophages Infected with *Shigella* Wild-type (A and D), caspase-1-deficient (B and E), or Ipaf-deficient BMMs (C and F) were infected with *Shigella* WT (A–C) or TTSS mutant (S325) (D–F) in the presence or absence of 3-MA (10 mM). Error bars represent mean ± SD. *, *p* < 0.05 (Mann–Whitney *U* test).

To begin to examine the functional role of autophagy in *Shigella*-induced cell death of infected macrophages, we incubated caspase-1- or Ipaf-deficient BMMs with 3-methyladenine (MA), a well known inhibitor of autophagy, after *Shigella* infection. Because 3-MA inhibits uptake of bacteria by macrophages [42], the compound was added after phagocytosis of bacteria by BMMs (10 min after infection). As shown in Figure 8A, the addition of 3-MA did not affect pyroptosis induced by *Shigella* infection in wild-type BMMs. Also, viability and multiplication of intracellular *Shigella* were not significantly affected by addition of 3-MA under microscopic observation (unpublished data). The treatment with 3-MA enhanced the LDH release from caspase-1- and Ipaf-deficient BMMs infected with *Shigella* (Figure 8B and 8C), suggesting that the inhibition of autophagy promotes *Shigella*-induced cell death in caspase-1- and Ipaf-deficient BMMs. In contrast, the addition of 3-MA did not affect LDH release from macrophages infected with the TTSS mutant (Figure 8D–8F), indicating that the cytosolic invasion is required for 3-MA to enhance membrane permeability associated with pyroptosis. These results suggest that autophagy induced by *Shigella* infection protect infected macrophages from pyroptosis.

## Discussion

Intracellular pathogenic bacteria trigger immune responses distinct from extracellular bacteria, which are mainly recognized by TLRs. Recent reports have indicated that cytosolic recognition of flagellin by *Salmonella* and *Legionella* mediates caspase-1 activation and IL-1β maturation [11,12,14,25,26]. The host protein Ipaf is required for activation of caspase-1 and IL-1β processing as well as for the inducement of rapid cell death through the sensing of intracellular flagellin during *Salmonella* and *Legionella* infection [11,12,14]. In this study, we demonstrate that Ipaf and its adaptor protein ASC are required for caspase-1 activation and IL-1β processing in *Shigella*-infected macrophages, but these processes, unlike in *Salmonella,* are independent of flagellin. The results suggest that unknown bacterial factor(s) are released from intracytosolic *Shigella* or secreted via the TTSS and are sensed directly or indirectly by Ipaf to promote caspase-1 activation. *Shigella*-induced activation of caspase-1 was previously attributed to IpaB [24]. Since IpaB is an integral component of the TTSS transmembrane pore complex that is inserted into the host cell membrane, the *ipaB* mutant is unable to translocate many effector proteins via TTSS. Thus, it is difficult or impossible to definitively attribute caspase-1 activation to IpaB alone by the use of the *Shigella ipaB* mutant. In *Salmonella,* the IpaB homolog SipB has been suggested to directly interact with caspase-1 and mediate its activation [43]. However, caspase-1 activation induced by *Salmonella* depends on the sensing of intracellular flagellin by Ipaf, but not SipB, in that flagellin mutants do not induce caspase-1 activation even though their SipB function is intact [11,12]. Because the TTSS in both *Shigella* and *Salmonella* forms a pore in the membrane of infected macrophages, the TTSS apparatus may induce a potassium ion efflux or another activity across the membrane, a signal that has been suggested as activating Nalp3 through the activation of the purigenic P2X7 receptor [6]. However, Nalp3 plays no function in caspase-1 activation induced by *Salmonella* infection [6] or *Shigella* infection (unpublished data). It was suggested that the recognition of intracellular flagellin by Ipaf is indirect [11], but the molecular mechanism by which flagellin is sensed by Ipaf is unclear. Thus, it is possible that both flagellin and the Ipaf-activating factor of *Shigella* interact with a common signaling machinery, and this host factor(s) is sensed by Ipaf to mediate caspase-1 activation. Further studies are needed to fully understand the molecular mechanism by which intracellular *Shigella* induces caspase-1 activation through the Ipaf-ASC-caspase-1 inflammasome.

The induction of caspase-1-independent pyroptotic cell death by *Shigella* infection was induced in Ipaf-deficient BMMs as well as in caspase-1-deficient BMMs [23]. We initially assumed that this phenotype was due to the lack of caspase-1 activity. However, ASC-deficient BMMs were not resistant to pyroptosis induced by *Shigella* despite the absence of caspase-1 activation. These results indicate that the function of Ipaf and ASC differ in a subtle manner and suggest that pyroptosis can proceed in the absence of caspase-1 activation. One possibility is that in caspase-1-deficient or Ipaf-deficient macrophages, anti-pyroptotic signals might be induced, leading to transient protection of macrophages from pyroptosis caused by bacterial infection. In this model, ASC might promote such a survival signal in the absence of caspase-1 or Ipaf. In certain experimental systems, ASC is known to mediate NF-κB activation [4,44], and thus NF-κB or another activity induced via ASC independently of caspase-1 might provide survival signals to counter the induction of pyroptosis in *Shigella*-infected macrophages.

We found that the induction of autophagy was facilitated by *Shigella* infection in the absence of caspase-1- or Ipaf-deficient BMMs but not in ASC-deficient BMMs. Because autophagy induced by amino acid starvation or by rapamycin treatment was normally induced in the absence of caspase-1 or Ipaf, the results indicate that the autophagic machinery is intact in the mutant cells and that caspase-1 activation inhibits autophagy formation in wild-type BMMs infected with *Shigella*. We hypothesized that, in wild-type macrophages, activated caspase-1 may degrade some factors that are essential for the induction of autophagy pathway, and that inhibition of autophagy and consequent rapid pyroptosis may serve to promote efficient induction of host inflammatory responses. Previous studies suggested that Naip5, another NLR family member, regulates autophagy in mouse macrophages infected with Legionella pneumophila [40]. The mechanism by which Naip5 and Ipaf/ASC/caspase-1 regulate autophagy in response to bacterial infection remains poorly understood. However, it is likely that these NLR proteins act through different mechanisms, as recent studies suggest that Ipaf, but not Naip5, controls caspase-1 activation [45]. The connection between caspase activation and autophagy are complex in that both events shared regulatory and mechanistic components [35]. Our results indicate that 3-MA, an inhibitor of autophagy, enhances cell death, thus raising the possibility that autophagy induction protects macrophages from cell death caused by *Shigella* infection. The mechanism by which *Shigella* or other intracellular bacteria trigger autophagy remains poorly understood. We have found no role for *Shigella* VirG in the induction of autophagy, in contrast to that reported in epithelial cells infected with *Shigella* [41]. It is likely that components released from intracellular bacteria into the host cytosol activate autophagy in that the *Shigella* TTSS mutant did not activate autophagy. Our results raise the possibility that caspase-1 activation and necrotic cell death, the two important activities for induction of pyroptosis after *Shigella* infection, represent independent phenomena. Our results also suggest that, at least in part, the delayed cell death observed in caspase-1-deficient BMMs may be a consequence of induction of autophagy. Further studies are needed to understand the molecular link between caspase-1 activation, pyroptosis, and autophagy, as well as their role in regulating host innate immune responses against intracellular bacteria.

## Materials and Methods

### *Shigella* strains and plasmids.

The wild-type S. flexneri 2a YSH6000 strain has been described previously [46]. *Shigella* mutants, S325 (*mxiA*::Tn*5*) [47], and a cell-to-cell spreading deficient *virG* null mutant (Δ*virG*) [41], were used in this study. The *fliC* mutant (Δ*fliC*) was constructed by allele replacement strategies according to the procedures described previously [48]. The wild-type S. enterica serovar Typhimurium SR-11 χ3181 and the isogenic *fliA*::Tn*10* were provided by H. Matsui (Kitasato Institute for Life Science, Tokyo, Japan) [49]. The *Shigella* and *Salmonella* strains were grown routinely in brain-heart infusion broth (Becton Dickinson, http://www.bd.com/) or Luria-Bertani broth, respectively. A *fliC* gene of *Shigella* was cloned downstream of the p*tac* promotor of expression vector pTB101-Tp [50]. FliC expression was driven by adding of 10 μM IPTG in the bacterial culture for 1 h.

### Reagents.

3-MA was purchased from Sigma (http://www.sigmaaldrich.com/). Anti-FliC antibody was provided from H. Matsui (Kitasato Institute for Life Science, Tokyo, Japan). The following antibodies were obtained commercially: rabbit anti-mouse caspase-1 (Santa Cruz Biotechnology, http://www.scbt.com/), goat anti-mouse IL-1β (R&D Systems, http://www.rndsystems.com/), and rabbit anti-LC3 antibody (MBL International, http://www.mblintl.com/).

### RT-PCR.

The bacteria grown to the exponential phase were stabilized by addition of RNAprotect bacteria reagent (Qiagen, http://www.qiagen.com/). Total RNA from the cells was prepared by using RNeasy mini kit and RNAse-free DNase (Qiagen) according to the protocols of the manufacturer, and converted to cDNA with ReverTra Ace (Toyobo Life Science, http://www.toyobo.co.jp/) as a template for PCR reactions. The primers for amplification of cDNA fragments were as follows: *fliC,* forward primer, 5′-CGTATTAACAGCGCGAAGGA-3′, reverse primer, 5′-AGACAGAACCTGCTGCGGTA-3′; *phoA,* forward primer, 5′-ATGTCACGGCCGAGACTTATAG-3′, reverse primer, 5′-GTGAATATCGACGCCCAGCG-3′; *ipaB,* forward primer, 5′-GCAGCAGTCGTTCTCGTAGC-3′, reverse primer, 5′-TCAAGCAGTAGTTTGTTGCAAAATTG-3′.

### Mice and preparation of macrophages.

BMMs were prepared from the femurs and tibias of caspase-1-deficient mice [23], Ipaf-deficient mice [11], and ASC-deficient mice [51] by culture for 5 d in 10% FCS-RPMI 1640 supplemented with 30% L-cell supernatant. All mice are C57BL/6 background or backcrossed with C57BL/6 mice. Mice were housed in a pathogen-free facility. Animal studies used protocols approved by the University of Michigan Committee on Use and Care of Animals (Ann Arbor, Michigan, United States), and the Animal Care and Use Committee of the Institute of Medical Science, University of Tokyo (Tokyo, Japan).

### Bacterial infection.

BMMs were seeded at 5 × 10^5^ cells in 24-well plates containing 10% FCS-RPMI 1640. The cells were infected with *Shigella* at an MOI of ∼10 per cell. The plates were centrifuged at 600*g* for 10 min to synchronize the stage of infection, and gentamicin (100 μg/ml) and kanamycin (60 μg/ml) were added 30 min later. At the times indicated after infection, the LDH activity of the culture supernatants of infected cells was measured by using a CytoTox 96 assay kit (Promega, http://www.promega.com/) according to the manufacturer's protocol. For immunofluorescence study, the infected cells were fixed and immunostained as described previously [23], and they were analyzed with a confocal laser-scanning microscope (LSM510; Carl Zeiss, http://www.zeiss.com/).

### Immunoblot.

BMMs seeded at 1 × 10^6^ cells in 6-well plates were infected with *Shigella* at an MOI of ∼10 per cell. Cells were lysed and combined with supernatants precipitated with 10% trichloroacetic acid. The samples were loaded onto 15% SDS-PAGE, and the cleaved form of caspase-1 and IL-1β were detected with anti-caspase-1 or anti- IL-1β antibody, respectively.

### Retroviral transfection.

Plat-E cells were transfected with pMX-puro-GFP or pMX-puro-GFP- rat LC3 using FuGENE 6 (Roche, http://www.roche.com/) [41,52]. Two-day cultures of BM cells were transfected with resulting retrovirus and cultured for an additional 3 d. In our experiments, GFP-transfected cells were 40%–50% and GFP-LC3-transfected cells were 30%–40% after recombinant virus infection, respectively. GFP and GFP-LC3 expression in BMMs were confirmed by the observation using a confocal laser-scanning microscope with the same threshold level. For induction of endogenous autophagy in amino acid–starved conditions, the BMMs were incubated with Earle's Balanced Salt Solution buffer (Sigma) for 2 h. To score autophagosome formation, a macrophages was defined as positive if it contained >10 donut-like shaped GFP-LC3-labeled structures. For induction of autophagy by rapamycin treatment, macrophages without retrovirus infection were incubated with rapamycin (25 μg/ml; LC Laboratories, http://www.lclabs.com/). At the indicated time, total cell lysates were prepared and analyzed by western blotting for detecting LC3-I to LC3-II conversion.

### Statistical analyses.

Statistical analyses were performed by the Mann–Whitney *U* test. Differences were considered significant at *p* < 0.05.

## Abbreviations

ASC: apoptosis-associated speck-like protein containing a C-terminal caspase recruitment domain
BMM: bone marrow–derived macrophage
IL: interleukin
LDH: lactate dehydrogenase
LPS: lipopolysaccharide
MA: methyladenine
MOI: multiplicity of infection
NLR: NOD-like receptor
NOD: nucleotide-binding oligomerization domain
PAMP: pathogen-associated molecular pattern
TLR: Toll-like receptor
TTSS: type III secretion system

## References

[ppat-0030111-b001] Janeway CA, Medzhitov R (2002). Innate immune recognition. Annu Rev Immunol.

[ppat-0030111-b002] Akira S, Uematsu S, Takeuchi O (2006). Pathogen recognition and innate immunity. Cell.

[ppat-0030111-b003] Inohara N, Chamaillard M, McDonald C, Nuñez G (2005). NOD-LRR proteins: Role in host-microbial interactions and inflammatory disease. Annu Rev Biochem.

[ppat-0030111-b004] Ting JP, Davis BK (2005). CATERPILLER: A novel gene family important in immunity, cell death, and diseases. Annu Rev Immunol.

[ppat-0030111-b005] Martinon F, Tschopp J (2004). Inflammatory caspases: Linking an intracellular innate immune system to autoinflammatory diseases. Cell.

[ppat-0030111-b006] Mariathasan S, Weiss DS, Newton K, McBride J, O'Rourke K (2006). Cryopyrin activates the inflammasome in response to toxins and ATP. Nature.

[ppat-0030111-b007] Kanneganti TD, Özören N, Body-Malapel M, Amer A, Park JH (2006). Bacterial RNA and small antiviral compounds activate caspase-1 through cryopyrin/Nalp3. Nature.

[ppat-0030111-b008] Martinon F, Petrilli V, Mayor A, Tardivel A, Tschopp J (2006). Gout-associated uric acid crystals activate the NALP3 inflammasome. Nature.

[ppat-0030111-b009] Sutterwala FS, Ogura Y, Szczepanik M, Lara-Tejero M, Lichtenberger GS (2006). Critical role for NALP3/CIAS1/Cryopyrin in innate and adaptive immunity through its regulation of caspase-1. Immunity.

[ppat-0030111-b010] Mariathasan S, Newton K, Monack DM, Vucic D, French DM (2004). Differential activation of the inflammasome by caspase-1 adaptors ASC and Ipaf. Nature.

[ppat-0030111-b011] Franchi L, Amer A, Body-Malapel M, Kanneganti TD, Özören N (2006). Cytosolic flagellin requires Ipaf for activation of caspase-1 and interleukin 1beta in salmonella-infected macrophages. Nat Immunol.

[ppat-0030111-b012] Miao EA, Alpuche-Aranda CM, Dors M, Clark AE, Bader MW (2006). Cytoplasmic flagellin activates caspase-1 and secretion of interleukin 1beta via Ipaf. Nat Immunol.

[ppat-0030111-b013] Zamboni DS, Kobayashi KS, Kohlsdorf T, Ogura Y, Long EM (2006). The Birc1e cytosolic pattern-recognition receptor contributes to the detection and control of Legionella pneumophila infection. Nat Immunol.

[ppat-0030111-b014] Amer A, Franchi L, Kanneganti TD, Body-Malapel M, Özören N (2006). Regulation of *Legionella* phagosome maturation and infection through flagellin and host Ipaf. J Biol Chem.

[ppat-0030111-b015] Mariathasan S, Weiss DS, Dixit VM, Monack DM (2005). Innate immunity against Francisella tularensis is dependent on the ASC/caspase-1 axis. J Exp Med.

[ppat-0030111-b016] Sansonetti PJ, Phalipon A, Arondel J, Thirumalai K, Banerjee S (2000). Caspase-1 activation of IL-1beta and IL-18 are essential for Shigella flexneri-induced inflammation. Immunity.

[ppat-0030111-b017] Maeda S, Hsu LC, Liu H, Bankston LA, Iimura M (2005). Nod2 mutation in Crohn's disease potentiates NF-kappaB activity and IL-1beta processing. Science.

[ppat-0030111-b018] Nadiri A, Wolinski MK, Saleh M (2006). The inflammatory caspases: Key players in the host response to pathogenic invasion and sepsis. J Immunol.

[ppat-0030111-b019] Suzuki T, Sasakawa C (2001). Molecular basis of the intracellular spreading of *Shigella*. Infect Immun.

[ppat-0030111-b020] Carlsson F, Brown EJ (2006). Actin-based motility of intracellular bacteria, and polarized surface distribution of the bacterial effector molecules. J Cell Physiol.

[ppat-0030111-b021] Fink SL, Cookson BT (2005). Apoptosis, pyroptosis, and necrosis: Mechanistic description of dead and dying eukaryotic cells. Infect Immun.

[ppat-0030111-b022] Brennan MA, Cookson BT (2000). *Salmonella* induces macrophage death by caspase-1-dependent necrosis. Mol Microbiol.

[ppat-0030111-b023] Suzuki T, Nakanishi K, Tsutsui H, Iwai H, Akira S (2005). A novel caspase-1/toll-like receptor 4-independent pathway of cell death induced by cytosolic *Shigella* in infected macrophages. J Biol Chem.

[ppat-0030111-b024] Hilbi H, Moss JE, Hersh D, Chen Y, Arondel J (1998). *Shigella*-induced apoptosis is dependent on caspase-1 which binds to IpaB. J Biol Chem.

[ppat-0030111-b025] Molofsky AB, Byrne BG, Whitfield NN, Madigan CA, Fuse ET (2006). Cytosolic recognition of flagellin by mouse macrophages restricts Legionella pneumophila infection. J Exp Med.

[ppat-0030111-b026] Ren T, Zamboni DS, Roy CR, Dietrich WF, Vance RE (2006). Flagellin-deficient *Legionella* mutants evade caspase-1- and Naip5-mediated macrophage immunity. PLoS Pathog.

[ppat-0030111-b027] Wei J, Goldberg MB, Burland V, Venkatesan MM, Deng W (2003). Complete genome sequence and comparative genomics of Shigella flexneri serotype 2a strain 2457T. Infect Immun.

[ppat-0030111-b028] Jin Q, Yuan Z, Xu J, Wang Y, Shen Y (2002). Genome sequence of Shigella flexneri 2a: Insights into pathogenicity through comparison with genomes of Escherichia coli K12 and O157. Nucleic Acids Res.

[ppat-0030111-b029] McClelland M, Sanderson KE, Spieth J, Clifton SW, Latreille P (2001). Complete genome sequence of Salmonella enterica serovar Typhimurium LT2. Nature.

[ppat-0030111-b030] Macnab RM (2003). How bacteria assemble flagella. Annu Rev Microbiol.

[ppat-0030111-b031] Chilcott GS, Hughes KT (2000). Coupling of flagellar gene expression to flagellar assembly in Salmonella enterica serovar typhimurium and Escherichia coli. Microbiol Mol Biol Rev.

[ppat-0030111-b032] Baehrecke EH (2005). Autophagy: Dual roles in life and death?. Nat Rev Mol Cell Biol.

[ppat-0030111-b033] Nakagawa I, Amano A, Mizushima N, Yamamoto A, Yamaguchi H (2004). Autophagy defends cells against invading group A *Streptococcus*. Science.

[ppat-0030111-b034] Ogawa M, Sasakawa C (2006). Bacterial evasion of the autophagic defense system. Curr Opin Microbiol.

[ppat-0030111-b035] Shintani T, Klionsky DJ (2004). Autophagy in health and disease: A double-edged sword. Science.

[ppat-0030111-b036] Swanson MS, Molofsky AB (2005). Autophagy and inflammatory cell death, partners of innate immunity. Autophagy.

[ppat-0030111-b037] Swanson MS (2006). Autophagy: Eating for good health. J Immunol.

[ppat-0030111-b038] Noda T, Ohsumi Y (1998). Tor, a phosphatidylinositol kinase homologue, controls autophagy in yeast. J Biol Chem.

[ppat-0030111-b039] Tanida I, Minematsu-Ikeguchi N, Ueno T, Kominami E (2005). Lysosomal turnover, but not a cellular level, of endogenous LC3 is a marker for autophagy. Autophagy.

[ppat-0030111-b040] Amer AO, Swanson MS (2005). Autophagy is an immediate macrophage response to Legionella pneumophila. Cell Microbiol.

[ppat-0030111-b041] Ogawa M, Yoshimori T, Suzuki T, Sagara H, Mizushima N (2005). Escape of intracellular *Shigella* from autophagy. Science.

[ppat-0030111-b042] Kirkegaard K, Taylor MP, Jackson WT (2004). Cellular autophagy: Surrender, avoidance and subversion by microorganisms. Nat Rev Microbiol.

[ppat-0030111-b043] Hersh D, Monack DM, Smith MR, Ghori N, Falkow S (1999). The *Salmonella* invasin SipB induces macrophage apoptosis by binding to caspase-1. Proc Natl Acad Sci U S A.

[ppat-0030111-b044] Masumoto J, Dowds TA, Schaner P, Chen FF, Ogura Y (2003). ASC is an activating adaptor for NF-kappa B and caspase-8-dependent apoptosis. Biochem Biophys Res Commun.

[ppat-0030111-b045] Lamkanfi M, Amer A, Kanneganti TD, Muñoz-Planillo R, Chen G (2007). The Nod-like receptor (NLR) family member Naip5/Birc1e restricts Legionella pneumophila growth independently of caspase-1 activation. J Immunol.

[ppat-0030111-b046] Sasakawa C, Kamata K, Sakai T, Murayama Y, Makino S (1986). Molecular alteration of the 140-megadalton plasmid associated with loss of virulence and congo red binding activity in Shigella flexneri. Infect Immun.

[ppat-0030111-b047] Watarai M, Tobe T, Yoshikawa M, Sasakawa C (1995). Contact of *Shigella* with host cells triggers release of Ipa invasins and is an essential function of invasiveness. EMBO J.

[ppat-0030111-b048] Datsenko KA, Wanner BL (2000). One-step inactivation of chromosomal genes in Escherichia coli K-12 using PCR products. Proc Natl Acad Sci USA.

[ppat-0030111-b049] Uematsu S, Jang MH, Chevrier N, Guo Z, Kumagai Y (2006). Detection of pathogenic intestinal bacteria by Toll-like receptor 5 on intestinal CD11c+ lamina propria cells. Nat Immunol.

[ppat-0030111-b050] Tobe T, Nagai S, Okada N, Adler B, Yoshikawa M (1991). Temperature-regulated expression of invasion genes in Shigella flexneri is controlled through the transcriptional activation of the virB gene on the large plasmid. Mol Microbiol.

[ppat-0030111-b051] Özören N, Masumoto J, Franchi L, Kanneganti TD, Body-Malapel M (2006). Distinct roles of TLR2 and the adaptor ASC in IL-1beta/IL-18 secretion in response to Listeria monocytogenes. J Immunol.

[ppat-0030111-b052] Suzuki M, Mimuro H, Suzuki T, Park M, Yamamoto T (2005). Interaction of CagA with Crk plays an important role in Helicobacter pylori-induced loss of gastric epithelial cell adhesion. J Exp Med.

